# Evaluating the effectiveness of a 'Tobacco Monitor' App in reporting violations of tobacco policy in the community

**DOI:** 10.6026/97320630017306

**Published:** 2021-02-28

**Authors:** Mehta Vini, Hegde S Sahana, Kakodkar Pradnya, Kumbhalwar Abhishek, Mathur Ankita

**Affiliations:** 1 Department of Public Health Dentistry, People's College of Dental Sciences & Research Centre, Bhopal, Madhya Pradesh; 2 Department of Public Health Dentistry, Dr. D.Y. Patil Dental College and Hospital, Pune; 3 D.Y. Patil Vidyapeeth, Pune; 4 Department of Public Health Dentistry, D.Y. Patil Dental School, Charholi, Lohegaon, Pune; 5 STAT SENSE, India

**Keywords:** Tobacco monitor app, violations, tobacco, smart phone apps

## Abstract

It is of interest to evaluate the effectiveness of the "Tobacco Monitor" app in reporting violations of tobacco policy in the community. Hence, a study was conducted amongst the first and second-year undergraduate students of health science colleges of a University. Students were asked to register complaints related to tobacco violations on the tobacco monitor app. Registered complaints were verified by the National Forum for Tobacco Eradication (NFTE) and descriptive statistics were used in reporting the results. A total of 208 complaints on tobacco violation were registered through the Tobacco Monitor app, 163 valid complaints were identified and 45 reports were found invalid. 163 verified valid complaints by NFTE were transferred to the Non-Communicable Diseases (NCD) Cell, Maharashtra, India. It should be noted that anti-tobacco laws and national policies help to curb the menace of the tobacco epidemic to an extent. However, robust reporting and sustainable enforcement measures are required in implementing tobacco legislation effectively. We also report that youth are comfortable in using the Tobacco Monitor app for reporting violations on tobacco.

## Background

With the increasing growth of new technology, smart phones have always been helpful in health promotion and health behaviour. From the Internet to email, they offer on-the-go access to information [1]. As a prelude to the World No Tobacco Day (WNTD) 2015, India's first-ever android app to report complaints on tobacco policy violations by users and tobacco industries was launched with the name "Tobacco Monitor" by the National Forum for Tobacco Eradication. (NFTE) The National Forum for Tobacco Eradication is a network comprising of individuals, professional associations, like-minded organizations, and institutions that are actively working towards tobacco eradication. NFTE closely works with various civil society members and Government officials for implementing tobacco eradication initiatives [2]. The new technology and a targeted app primarily bestowed three benefits that can be useful: Firstly, people can utilize this app effectively to create a tobacco- free environment. Secondly, it also provides information and support for tobacco eradication. Lastly, the app majorly aims to brace Government motives by facilitating evidence-based complaints and helps them to take necessary action against the violators [3]. Smart phones offer low cost, convenient, and faster [4] ways to register complaints. The user can click a picture of the violation and send it as proof. Upon submission of the complaint, the NFTE receives it, and later reviews it. Once the complaint is verified it will be sent to the State Tobacco Control Cell, the Concerned Departments of the Respective State, and Civil Society Members who are registered with NFTE. A global public health treaty, namely the Framework Convention on Tobacco Control (FCTC) was negotiated to address the growing menace of tobacco [5]. India played an active role on the tobacco control front by enacting the Cigarettes and other Tobacco Products (Prohibition of Advertisement and Regulation of Trade and Commerce, Production, Supply, and Distribution) Act, 2003 (COTPA), which covered all tobacco products [6]. This law was intended to safeguard and protect public health, by making provisions of evidence-based strategies to reduce tobacco consumption [7]. Therefore, it is of interest to evaluate the effectiveness of the "Tobacco Monitor" app in reporting violations of tobacco policy in the community.

## Methodology

A study was conducted amongst the first and second-year undergraduate students of health science colleges of a University. The target population consisted of students from Dental, Homeopathy, Physiotherapy, Nursing, and Ayurvedic streams. Only those students who gave informed consent, having android phones, willing to download the app, and were willing to report violations on tobacco policies through the Tobacco Monitor app were included. Ethical clearance was obtained from the Institutional Ethics Committee. A consent form was obtained from NFTE stating that they are willing to provide all the required information for the concerned research project.

## Data Collection:

The tobacco monitor app was available for android and iOS users. Mary Anne Charity Trust (MACT), Chennai, developed the App. Tobacco Monitor App is a platform where a person can acquire information about the latest happenings related to tobacco eradications, regulations, and cessation, and also give complaints on tobacco-related violations. The app works at two levels, namely as a platform to obtain the latest updates on tobacco eradication and to request referral services. At the second level, a user can report any violations of tobacco. Assessing the app in monitoring the Tobacco Industry's activities, especially regarding their violation of Tobacco Control Laws, Policies, Orders such as WHO - Framework Convention on Tobacco Control (FCTC), Cigarette and Other Tobacco Products Act (COTPA), 2003 and challenging the violators. There were 28 different violation types for the user interface. User anonymity and confidentiality of the information provided was assured. The status of the registered complaint was provided to the user. A batch of 1000 students was addressed at a time in the classroom. A PowerPoint presentation of 30 minutes duration on tobacco control legislation in India was presented to them. A video of 10 minutes was shown regarding the use of the Tobacco Monitor app along with the instructions about downloading the app on their smartphones. Students were asked to register complaints with intricate details such as their mobile number, organization name/violating shop, date, location, state, district, street name, city, type of violation, and the picture of violation while submitting any instance or complaint. Students were instructed to identify themselves on the app with their University name to facilitate study exclusive information retrieval and anonymity. The remark section allowed users to describe the violation. Registered complaints were verified by NFTE with the help of an authenticator application. An attempt was made by NFTE to recollect the information in case of incomplete details. A reinforcement message about the use of the Tobacco Monitor app was sent at the end of 1 month via SMS to the respective students. At the end of three months, the data from NFTE regarding tobacco violations were obtained and descriptive statistics were used in reporting the results.

## Results:

**Figure 1:**
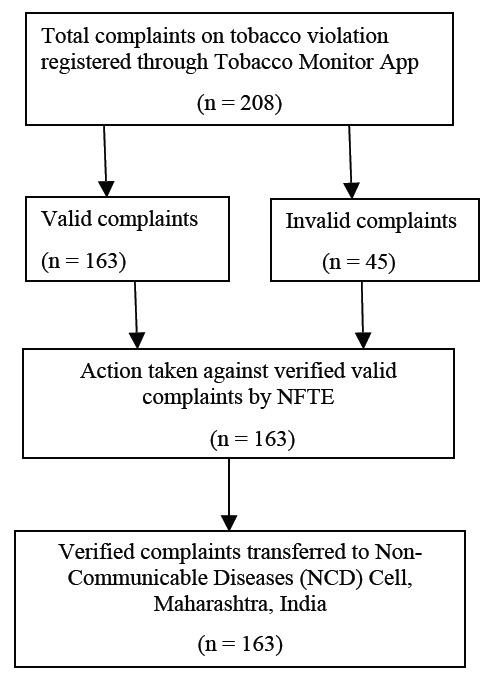
Flowchart summarizing the status of complaints

A total of 360 students downloaded the "Tobacco Monitor" app. Overall, 208 complaints on tobacco violation were registered through the Tobacco Monitor app, 163 valid complaints were identified, and 45 were invalid. 163 verified valid complaints by NFTE were transferred to the Non-Communicable Diseases (NCD) Cell, Maharashtra, India. Pictures were attached in the form of evidence in all complaints (Figure 1). Out of 163 violations, 44.17% of the violations consisted of tobacco product advertisements, 28.9% of the violations were registered under the sale of Pan Parag and Gutka. 12.88% were reported for the Point of sale violation. Sale within 100 yards of educational institutions consisted of 3.68% and 2.49%of sale was registered as loose tobacco products. The presence of hookah bars and advertisements in the media consisted of 0.61% of the total sale (Table 1). Major tobacco violations i.e. 45.4% were seen in the market area, 39.9% in petty shops, 4.9% in tea shops and bus stand. The distribution of violations in schools and hospitals was 3% and 0.7% respectively (Table 2).

## Discussion:

Youngsters have rapidly adapted smartphones in everyday life. Downloads of mobile health care applications have nearly doubled from 127 million in 2011 to 247 million in 2015 [8]. The widespread use of mobile technologies propounds innovative ways to improve public health. Teaching about tobacco violations and their related consequences is essential for undergraduate students, especially to counter the adverse effects of tobacco. The healthcare undergraduate students were recruited in the present study. It has been reported that health professionals have a vital role to fight against the tobacco epidemic and they can influence national and global tobacco control efforts by educating people who can support anti-smoking policies at a societal level [9]. Physicians can also play an important role in leading smoking cessation campaigns [10]. In the present study, the "Tobacco monitor" app reported the highest percentage of violations for tobacco product advertisements (44.17%) and point of sale (12.88%). Similar results were obtained in other studies, [11-14] wherein the violations of point of sale advertisements were the size of aboard, backlit, brand name/packshot, display of the promotional message, and advertisements extended to full body. The remark section in the app described most of the point of sale violations. Literature reveals that tobacco advertisements account for 45-80% of complaints thus violating the legal provision of section 5 [14,15]. It is evident from the results that the Indian tobacco industry has taken advantage of the loophole in COTPA, which allows advertising. Attractive show casings dangle, and stickers, which are not permitted under the law, were used for advertising which is evident from the attached picture in the Tobacco Monitor app. In the present study, market place accounts for 45.4% of violations. Manufacturers pay for point of sale advertisements and usually, these advertisements are located at the busy market places or on important roadsides measures up to the fact that these are also meant for sales promotion and not just for providing information about the availability of products. The government of Maharashtra banned the sale of Pan Parag and Gutka and classified them as food to come under the purview of the Food Safety and Standards Act, 2006. In a survey, 41% of tobacco outlets were displaying and selling Pan Parag packets and Gutka. [16] From the registered complaint, Pan Masala scented tobacco was seized from Siddheshwar Panwala, Shivar Garden Road, Pimple Saudagar, Pune, and action was taken against the vendors regarding violation of COTPA 2003. Smoke-free legislation under section 4 has various parameters to assess compliance. It was found that active smoking was observed at 6.74%. Most frequently visited public places showed the highest frequency of violation in terms of smoking, however, reporting with evidence-based pictures are difficult to click. Petty shops, tea shops, hotel/restaurant, and bus stands observed 49.7% of violations. Better compliance under section 4 can be achieved with increased public awareness, media support, and strict enforcement of the law. In the present study, tobacco sale within 100 yards of educational institutions accounts for 3.68%. Furthermore, a ban on the use of tobacco forms a component of a healthy school environment according to WHO based health promoting schools framework [17] and this initiative has been proved to be successful in Tamil Nadu [18]. Exposure to tobacco products at an early age results in the initiation of smoking at an older age [19]. 2.49% of the complaints reported on the app were regarding the sale of loose tobacco products, which is based on COTPA laws. Loose Cigarettes and Bid is being banned in Maharashtra under the Legal Metrology Act, 2009 [20]. All the reported complaints had an attached picture, which gave consolidated evidence against violations. Most of the photographs gave a clear picture of violations. Similarly, a survey [21] collected photographs as evidence of potential or actual violations. Kaur et al. [22] highlighted that ensuring a reviewable reporting system plays a pivotal role in driving COTPA. A robust reporting and monitoring system can help the government to take necessary actions such as collecting fine deposits, printing challan, and imposing penalties.

After verification of 208 registered complaints, 45 were found invalid due to insufficient information or duplicate data. Some of the complaints lacked comprehensive details and hence no action was taken against these complaints. However, an attempt was made to recollect the data from the participants by calling them. All the verified complaints were transferred to Non-Communicable Diseases (NCD) Cell, Maharashtra, India by NFTE. Right to Information (RTI) was filed against the Information officer, District Civil Surgeon, District Civil Hospital, Aundh to request the government to take strict action against all the complaints. The present study thus provides information that the younger generation is the greatest asset of our country, who can provide concrete evidence on tobacco violations. The efficacy of the apps would depend on how individuals make use of them. There is a dire need for comprehensive training for all health science students to demolish the tobacco menace. Reporting policy violations builds positive attitudes, which in turn benefits the community in multiple ways. Effective implementation of anti-tobacco laws remains a huge challenge. Health science students have an important role to play in fighting this challenge. As doctors interface with the community, they can significantly contribute to curbing the tobacco epidemic. Tobacco violations are socially and culturally unaccepted and hence students might have selectively underreported. App-specific limitations have to be explored for unleashing its fullest potential. Various enforcing agencies should be involved in each stage to implement the act effectively. Current data findings can be used to identify lacunae in tobacco policy violations to facilitate effective implementation. The effectiveness of any novice technologies like apps would depend on how individuals make use of them. There is a dire need for comprehensive training for all health science students to demolish the tobacco menace. Reporting policy violations builds positive attitudes, which in turn benefits the community in multiple ways.

## Conclusion

Anti-tobacco laws and national policies are helping to an extent in curbing the menace of the tobacco epidemic. However, robust reporting and sustainable enforcement measures are required in implementing tobacco legislation effectively. Data shows that the youth effectively used the Tobacco Monitor app in reporting violations about tobacco. This provides an opportunity to enforce government agencies and policymakers to take adequate actions against the registered complaints.

## Figures and Tables

**Table 1:** Distribution of violations according to Tobacco Monitor app.

Different kind of violations	Number (Reported)	Percentage (%)
Smoking in Public Place (Section 4)	11	6.74
Presence of Hookah Bar (Section 4)	1	0.61
The Prohibition of Smoking in Public Places Rules, 2008		
Tobacco Products Advertisement (Section 5)	72	44.17
Point of Sale (Section 5)	21	12.88
Indirect Advertisement (Section 5)	1	0.61
Sale within 100 Yards (Section 6)	6	3.68
Sale of Loose Tobacco Products (Legal Metrology Act)	4	2.49
Sale of Pan Parag, Gutka (Food Safety and Standards Act, 2006)	47	28.9
Total	163	100

**Table 2:** Distribution of location according to Tobacco Monitor app.

Location	Number	Percentage (%)
Petty Shop	65	39.9
Tea Shop	8	4.9
Hotel/Restaurants	2	1.2
Bus Stand	8	4.9
School	5	3
Market	74	45.4
Hospital	1	0.7
Total	163	100
